# Adipose Tissue Metabolism in Response to Food Intake

**DOI:** 10.3390/nu15224811

**Published:** 2023-11-17

**Authors:** Andres E. Carrillo, Maria Vliora

**Affiliations:** 1 Department of Exercise Science, School of Health Sciences, Chatham University, Pittsburgh, PA 15232, USA; 2 FAME Laboratory, Department of Physical Education and Sport Science, University of Thessaly, 42100 Trikala, Greece; mvliora@gmail.com

The quality and quantity of the food we consume have a major impact on our general health and longevity. Western dietary trends include an abundance of calories and refined sugars that contribute to adipose tissue accumulation, dysregulated adipose tissue function, and an increased risk of chronic disease [1]. Adipose tissue, once regarded simply as an energy reservoir, is now recognized as a dynamic organ that finely tunes its metabolic activities in response to various dietary inputs [2]. This Special Issue in *Nutrients*, “Food Intake and Adipose Tissue Metabolism” aims to create a collection of scientific papers that explore how the relationship between food intake and adipose tissue metabolism influence general health, including chronic disease risk and longevity. Furthermore, enhancing our ability to navigate the complex interactions existing between food intake and adipose tissue has important implications for public and personalized health.

The macronutrient composition of food has a strong influence on adipose tissue metabolism. High-carbohydrate diets, especially those rich in refined sugars, can lead to insulin resistance and adipose tissue inflammation. Conversely, diets emphasizing unsaturated fats and whole grains promote adipose tissue health [1]. Proteins, through their thermogenic effect and role in muscle preservation, influence adiposity and metabolic rate. Other studies have investigated particular food compounds that have been shown to influence adipocyte function and chronic disease risk. For example, an article published by Nakadate et al. [3] in this Special Issue investigates adipocyte size and function in obese mice following a four-week treatment period of tea catechin and citrus β-cryptoxanthin—a citrus-derived polyphenol. The authors report an overall reduction in adipocyte size, reduced inflammation (i.e., tumor necrosis factor alpha, interleukin six), and normalized adiponectin following the treatment period, concluding that the combined ingestion of tea catechin and citrus β-cryptoxanthin improves adipocyte function.

To further advance our understanding of the relationship between food intake and adipose tissue, it is important to consider the concept of naïve substitution proposed by Waters [4]. Naïve substitution emphasizes the importance of precision with language in guiding scientific discovery, the interpretation of new information, and informing clinical practice. In other words, not all adipose tissue is created equal. White adipose tissue (WAT)—mostly located in subcutaneous and visceral fat depots—provides an efficient site for energy storage, contains few mitochondria, and interacts with other cells and tissues via secreted hormones and inflammatory markers [5]. Subcutaneous fat accumulation, however, appears to be considerably less lethal when compared to the more metabolically active visceral fat that surrounds internal organs [6,7]. Visceral fat accumulation has been associated with increased macrophage infiltration of adipose tissue, resulting in chronic inflammation and a greater risk of cardiovascular disease and metabolic dysfunction [8]. Data from the PREDIMED-Plus study revealed clinical improvements in circulating inflammatory markers, adipocyte function, and circulating insulin in response to diet-induced visceral fat loss in patients with metabolic syndrome [9].

Brown adipose tissue (BAT) is another classification of adipose tissue that accumulates above the clavicle and in the posterior subscapular region but typically declines with advancing age [10]. Brown adipose tissue is enriched with mitochondria that function to transfer potential energy from food to heat energy via the uncoupling protein 1 (UCP-1)—a protein embedded within the inner mitochondrial membrane that uncouples heat production from adenosine triphosphate synthesis to maintain thermal homeostasis. A greater BAT depot in adults has been associated with improved metabolic function and a lower body mass index. Interestingly, a study conducted in mice showed that insulin treatment resulted in reduced UCP-1 in BAT that contributed to a reduction in mitochondrial respiration [11]. In another study, a ketogenic diet fed to mice for one month resulted in reduced insulin concentrations, increased mitochondrial size, and elevated UCP-1 levels in BAT when compared to control mice [12]. Beige adipocytes are a unique lipid subtype with characteristics of both white and brown adipose tissue mostly located in subcutaneous fat. Similar to BAT, the browning of WAT or accumulation of beige adipose tissue has been associated with improved metabolic function [13]. A few studies have found that diet can induce browning [14], while others have shown contradictory results [15]. For example, in humans, browning formation markers such as peroxisome proliferator-activated receptor gamma coactivator 1-alpha and UCP-1, measured from subcutaneous fat biopsies, were not associated with diet in healthy adult men [15].

Bone marrow adipose tissue (BMAT) is found mostly in long bones, vertebrae, and the iliac crest. This unique classification of adipose tissue displays characteristics of both WAT and BAT; however, the exact function of BMAT remains uncertain [16]. In fact, recent studies have suggested that BMAT has metabolic functions that respond to nutritional challenges and impact energy and bone metabolism [17,18]. Accumulation of BMAT has been shown to occur with advancing age and appears to follow a U-shaped curve in relation to total body fat [18]. Specifically, Newton et al. [19] assessed body composition in 59 early-pubertal girls and found a positive association between body fat and BMAT. Another study conducted in Latino adolescents found that greater BMAT content was positively associated with liver fat and visceral adipose tissue [20]. Conversely, BMAT accumulation was negatively correlated with bone strength in anorexia nervosa patients [21]. Fazeli et al. [18] reported differences in BMAT immune responses induced by high calorie feeding compared to fasting that may reflect what occurs in peripheral adipose tissue.

To summarize, there is considerable complexity in adipocyte function between and within adipose tissue classifications that are influenced by body composition and nutritional challenges. As we delve deeper into the molecular processes governing these interactions, novel therapeutic strategies and dietary interventions will undoubtedly emerge. This Special Issue aims to collate scientific papers that contribute to further understanding how adipocytes from diverse adipose tissue classifications function in response to different nutrients and/or dietary strategies with the intention of moving in the direction of a healthier future.
